# The impact of general and central obesity for all-cause hospitalization among Iranian adults: a 20 year follow-up-results from the TLGS cohort

**DOI:** 10.1186/s12889-023-15851-0

**Published:** 2023-05-18

**Authors:** Azra Ramezankhani, Fereidoun Azizi, Mitra Hasheminia, Farzad Hadaegh

**Affiliations:** 1 Prevention of Metabolic Disorders Research Center, Research Institute for Endocrine Sciences, Shahid Beheshti University of Medical Sciences, Floor 3th, Number 24, Yemen Street, Shahid Chamran Highway, P.O. Box: 19395-4763, Tehran, Iran; 2 Endocrine Research Center, Research Institute for Endocrine Sciences, Shahid Beheshti University of Medical Sciences, Tehran, Iran

**Keywords:** BMI, Waist circumference, Obesity, Hospitalization, Cardiovascular disease

## Abstract

**Background:**

Few studies have examined the effect of obesity indices on total number of hospitalizations. We examined the associations between body mass index (BMI) and waist circumference (WC) and rate of all-cause hospitalizations among Iranian adult participated in the Tehran Lipid and Glucose Study cohort.

**Methods:**

This study included 8202 individuals (3727 men) aged ≥ 30 years, who were followed for a median of 18 years. Participants were categorized into three groups according to their baseline BMI: normal weight, overweight and obese. In addition, they were classified according to WC in two categories: normal WC and high WC. Negative Binomial regression model was used to estimate the incidence rate ratios (IRRs) and 95% confidence interval (95% CI) of all-cause hospitalizations in relation to obesity indices.

**Results:**

The overall crude rate of all-cause hospitalizations were 77.6 (95% CI, 73.9–81.2) and 76.9 (73.4–80.3) per 1000 person-year in men and women, respectively. The covariate adjusted rate of all-cause hospitalizations was 27% higher in obese men than normal weight men (IRR (95% CI): 1.27 (1.11–1.42)). Among women, overweight and obese individuals had 17% (1.17 (1.03–1.31)) and 40% (1.40 (1.23–1.56)) higher rate of hospitalization compared to normal weight women. Having high WC was associated with 18% (1.18 (1.08–1.29)) and 30% (1.30 (1.18–1.41)) higher rate of all-cause hospitalization in men and women, respectively.

**Conclusions:**

Obesity and high WC were associated with increased hospitalization rates during long-term follow-up. Our findings suggests that successful obesity prevention programs may decrease the number of hospitalizations, particularly, in women.

**Supplementary Information:**

The online version contains supplementary material available at 10.1186/s12889-023-15851-0.

## Background

Obesity is a complex chronic disorder [1] and lead to poor health and increased risk of numerous chronic diseases, mortality and social costs [2]. According to Global Burden of Disease (GBD) study in 2017, high body mass index (BMI) increased by 127% between 1990 and 2017 [3]. Although the prevalence of high BMI has increased globally, the rise is more profound in the Middle East and North Africa (MENA) region [4]. In 2016, a national study in Iran reported that the prevalence of overweight/obesity was 59.3% among adults aged ≥ 18 years [5].

The global increase in BMI has far-reaching implications for the population health and wellbeing. In 2019, the GBD study reported that 5.02 million deaths and 160 million disability-adjusted life-years were attributable to high BMI [6]. In 2016, 47% of the total cost of all chronic diseases was due to high BMI among US adults [1].

While there is considerable studies supporting the relation between obesity and the health outcomes, the evidence on the risk of hospitalization in relation to obesity indices is less clear. Several studies have examined the impact of BMI on the risk of first hospitalization, with the mixed results; some studies found positive associations [7–9] and others found no association or negative association [10, 11]. Moreover, only two studies have examined the effect of overweight/obesity on total number of hospitalizations which better reflects the cumulative burden of increased hospitalization associated with elevated level of BMI [12, 13].

To date, no such study has been conducted in the MENA region, despite increasing prevalence of obesity in this region [14]. Therefore, for the first time, we examined whether obesity indices including BMI and waist circumference (WC) were associated with total number of all-cause hospitalizations over a median of 18 years of follow-up among Iranian adults using longitudinal data from the Tehran lipid and glucose study (TLGS) cohort.

### Methods

The TLGS is a population-based prospective investigation of the prevalence and incidence of non-communicable diseases (NCD) and their risk factors conducted in district 13 of Tehran, capital of Iran. The details of the study have been explained previously [15]. Briefly, in Phase 1 (1999–2001), a total of 15,005 individuals aged ≥ 3 years were recruited, and then, 3550 new subjects were included in Phase 2 (2002–2005), bringing the total study population to 18,555 individuals. Baseline data were collected using the questionnaire interviews, clinical examinations, and laboratory assessments. All participants were followed up triennially since enrolment [16]. For this study, we included 9558 participants ≥ 30 years from phase 1 (n = 7927) and phase 2 (n = 1631) and excluded those without any follow-up data after baseline recruitment (n = 879), those with missing data on BMI and WC status (n = 276), underweight participants (BMI < 18.5 kg/m^2^) (n = 109), and those with missing data on other covariates (n = 92), leaving us 8202 individuals (3727 men) (more than 85% of eligible participants) who were followed up until the end of the study (20 march 2018) (Supplementary Fig. 1). Informed consent were obtained from all participants. This study was approved by the ethical committee of the Research Institute for Endocrine Sciences of Shahid Beheshti University of Medical sciences.

### Measurement

Anthropometric measures including weight, height and WC were recorded using standard protocols [15]. Weight was measured using digital scales and height was measured in a standing position. WC was measured at the umbilical level using a tape meter. Physical activity level (PAL) was assessed by the Lipid Research Clinics questionnaire [17] in Phase 1, and Modifiable Activity Questionnaire in Phase 2 [18]. Marital status was defined as: single, married, widowed/divorced. Educational level was categorized as: <6 years, 6–12 years, and > 12 years of schooling. Smoking status was categorized as current smoker, past-smoker and never smoker. A current smoker was a person who smokes cigarettes or other smoking implements daily or occasionally. Never smokers included people who had never smoked, and past smokers were defined as having quit smoking for at least 1 year prior to study enrolment. PAL was categorized as high and low. In Phase 1, high PAL was defined as doing exercise or labor ≥ 3 times/week, and in Phase 2, it was defined as achieving a score ≥ 600 MET (metabolic equivalent task minutes)/week [19]. BMI was calculated as weight in kilograms divided by height in square meters (kg/m^2^), and were categorized in three groups: normal weight (BMI ≥ 18.5 to < 25.0 kg m^2^), overweight (BMI ≥ 25.0 to < 30.0 kg m^2^) and obese (BMI ≥ 30.0 kg m^2^). According to proposed WC cut-offs for the Iranian adult population at risk of cardiovascular disease (CVD) events [20] we classified WC in two categories: normal WC (< 95 cm) and high WC (≥ 95 cm) for both sexes.

### Assessment of outcome

The main outcome was total number of all-cause hospitalizations per follow-up time. Hospitalization was defined as admission to a hospital for any reason at least one night. All cohort members were followed up annually for any medical event leading to hospitalization during the past year, by telephone call to them or their family [16]. Then, a trained physician collected related data through hospital records. Data were further reviewed by an expert committee. In this committee, the primary cause for hospitalization was defined using the first International Classification of Diseases, 10th revision (ICD-10) code. We excluded the hospitalizations for the caesarian section, normal vaginal delivery, cosmetic surgeries, and laser therapy for diabetic retinopathies. Additionally, we categorized the primary causes into 9 specific diagnosis groups: coronary heart disease (CHD), stroke, infectious disease, respiratory disease, type 2 diabetes mellitus (DM) complications, hypertension complications, neoplasm, traumas, and others (Supplementary Table 1). Follow-up time was defined as the interval (years) between enrollment and end of study (20 March 2018), date of death or date of loss to follow-up, whichever came first. Participants who died during the study period were included in the analyses, as the aim of this study was to assess the effect of baseline risk factors on the total number of hospitalizations over observation period.

### Statistical analysis

Baseline characteristics of the study population are presented for men and women. To compare means or percentages of variables we used one-way analysis of variance (ANOVA) and chi-squared tests, respectively. In addition, we compared baseline characteristics between participants and non-participants using t-test and chi-squared tests. Non-participants were those who excluded from the study due to having missing data on BMI, WC and other covariates at baseline and people without any follow-up data after enrolment to study.

We estimated the crude incidence rates of all-cause and cause specific hospitalization per 1000 person-years with 95% confidence interval (95% CI) in total population, and in men and women, separately. Moreover, crude incidence rate ratios (IRRs) (95% CI) of all-cause hospitalization were computed by BMI and WC categories in men and women. To estimate the adjusted IRRs we used the negative binomial (NB) regression model with robust standard errors. We selected the NB model over a Poisson model due to the presence of overdispersion in the outcome, which was confirmed by the significant likelihood ratio test (P < 0.001) for the null hypothesis of no overdispersion. Adjustments in NB models were made for potential confounding variables. In model 1, we adjusted for age, and model 2 was further adjusted for education level, smoking status, marital status and PAL. We did not adjust for comorbidities such as diabetes, hypertension and CVD that potentially mediate the effects of overweight/obesity on hospitalizations.

In all regression models, follow-up time was included as an offset variable to account for varying follow-up times (due to death or lost to follow-up) as well as the competing risk of death, as used in previous studies [21–23].

The coefficients of NB models were then used to calculate adjusted rates of hospitalization in different groups of BMI and WC. In fact, adjusted rates are predicted outcome based on the NB models for the specified values of the predictor variables. We estimated adjusted rates in a representative value by setting the continuous variables to the mean and categorical variables to the reference category. Continuous variable included age and categorical variables included smoking status (never [reference]), education status (< 6 years [reference]), marital status (married [reference]) and PAL (low [reference]). Moreover, absolute rates based on the age-adjusted NB model (model 1) were plotted over the age range in men and women, separately.

In our study, about 46% of population was never hospitalized during their follow-up; therefore, in a sensitivity analyses, we developed multivariate zero-inflated NB models to calculate adjusted rates of hospitalization in men and women. Analyses were performed using IBM SPSS Statistics version 20 (IBM Corp) and R version 4.1.2. In all analyses, a two-sided P-value < 0.05 was considered statistically significant.

## Results

The baseline characteristic of participants and non-participants are shown in Supplementary Table 2. Participants were more likely than non-participants to have high PAL and to be never smoker; whereas, they had higher levels of BMI and WC than non-participants.

However, in both participant and non-participant groups, the mean of BMI in people with high PAL were lower than people with low PAL (data not shown).

Study sample (participants) included 3227 men and 4475 women. The mean age of men and women were 48.1 and 46.3 years, respectively. About 63% of the men and 77.6% of women were overweight/obese. Further, 38.0% and 38.5% of men and women, respectively, had high WC. The baseline characteristic of participants are described in Table 1. In both sexes, the mean of BMI and WC were higher in obese than normal weight individuals, as expected. The proportion of low educational level increased with increasing BMI in both men and women. The obese women had lower PAL compared with normal weight women, and the obese men were less likely to be current smoker than normal weight men.

**Table 1:** Baseline characteristics of participants by BMI category and sex: the Tehran lipid and glucose study, 1999–2018

	Men n = 3727					Women n = 4475			
	Normal (n = 1389)	Overweight (n = 1737)	Obese (n = 601)	P value		Normal (n = 999)	Overweight (n = 1867)	Obese (n = 1609)	P value
**Continuous variables**									
Age (years)	48.4 (13.8)	48.4 (12.7)	47.6 (12.5)	0.359		44.1 (12.7)	46.8 (11.6)	48.0 (10.6)	< 0.001
BMI (kg/m^2^)	22.6 (1.7)	27.2 (1.3)	32.4 (2.5)	< 0.001		22.8 (1.5)	27.5 (1.4)	33.6 (3.3)	< 0.001
Waist circumference (cm)	82.1 (6.7)	93.8 (6.1)	105.0 (7.5)	< 0.001		78.4 (7.3)	89.1 (7.8)	101.2 (9.1)	< 0.001
**Categorical variables**									
**Smoking (%)**									
Never	666 (47.9)	936 (53.9)	348 (57.9)	< 0.001		931 (93.2)	1727 (92.5)	1491 (92.7)	0.193
Past	217 (15.6)	302 (17.4)	102 (17.0)			16 (1.6)	56 (3.0)	38 (2.4)	
Current	506 (36.5)	499 (28.7)	151 (25.1)			52 (5.2)	84 (4.5)	80 (5.0)	
**Marital status (%)**									
Single	82 (5.9)	49 (2.8)	16 (2.7)	< 0.001		99 (9.9)	41 (2.2)	31 (1.9)	< 0.001
Married	1291 (92.9)	1672 (96.3)	580 (96.5)			788 (78.9)	1588 (85.1)	1348 (83.8)	
Widowed/divorced	16 (1.2)	16 (0.9)	5 (0.8)			112 (11.2)	238 (12.7)	230 (14.3)	
**Educational level (%)**									
< 6 years	411 (29.6)	520 (29.9)	205 (34.1)	0.048		377 (37.7)	868 (46.5)	919 (57.1)	< 0.001
6–12	718 (51.7)	906 (52.2)	314 (52.2)			500 (50.1)	864 (46.3)	623 (38.7)	
≥ 12	260 (18.7)	311 (17.9)	82 (13.6)			122 (12.2)	135 (7.2)	67 (4.2)	
**Physical activity level**									
High	414 (29.8)	495 (28.5)	168 (28.0)	0.621		316 (31.6)	631 (33.8)	475 (29.5)	0.026
Low	975 (70.2)	1242 (71.5)	433 (72.0)			683 (68.4)	1236 (66.2)	1134 (70.5)	

**BMI**: body mass index; **Normal weight**: BMI ≥ 18.5 to < 25.0 kg m^2^; **Overweight**: BMI ≥ 25.0 to < 30.0 kg m^2^; **Obese**: BMI ≥ 30.0 kg m^2^

### Crude estimates

Among 8202 total participants and during 127,431 person-year of follow-up with the median and interquartile range (IQR) of 18.1 (15.1–18.5) years, 1012 deaths (600 men) were identified. During study period, a total of 9840 all-cause hospitalizations occurred, with an overall crude rate of 77.2 (74.7–79.7) per 1000 person-year in total participants, 77.6 (73.9–81.2) in men, and 76.9 (73.4–80.3) in women.

In total participants, the crude rates of all-cause hospitalization were 73.4 (68.5–78.3) in normal weight, 79.7 (75.8–83.7) in over weight and 95.1 (89.5-100.6) per 1000 person-year in obese individuals (supplementary Table 3). The corresponding values by WC categories were 64.8 (62.1–67.6) and 97.9 (93.2-102.6) per 1000 person-year in those with normal and high WC, respectively (Supplementary Table 4).

The crude rates of all-cause and cause-specific hospitalization in men and women by BMI and WC categories are presented in Supplementary Tables 5 and 6, respectively. Overall, in both sexes, among known causes of hospitalization, CHD was the major cause of hospitalization, with the higher rate in men than women. Moreover, there was evidence that crud rate of all-cause hospitalization, CHD- and stroke-related hospitalization and hospitalization attributed to DM complications was greater in obese women than normal weight women (Supplementary Table 5). Having high WC, was associated with greater crude rate of all-cause hospitalization and CHD- and stroke-related hospitalization in men. However, in women, it was associated with all-cause hospitalization and all specific causes of hospitalization except for cancer (Supplementary Table 6).

### Adjusted estimates

The crude rate of all-cause hospitalization was 17% higher (RR (95% CI): 1.17 (1.07–1.27)) in obese men than normal weight men (Table 2). After adjustments for age, and other covariates including education level, smoking status, marital status and PAL, obese men had 27% more hospitalizations rate than normal weight men (1.27; 1.11–1.42). Among women, both overweight and obese individuals had higher crude rate of hospitalization compared to normal weight women (1.33 (1.22–1.43) and 1.67 (1.55–1.81), respectively). After adjustments for age and other covariates, overweight and obese women had a higher risk of hospitalization than normal weight women (1.17 (1.03–1.31) and 1.40 (1.23–1.56), respectively). Moreover, in multivariate adjusted models, men and women with high WC had 18% (1.18; 1.08–1.29) and 30% (1.30; 1.18–1.41), respectively, higher rate of hospitalization than their normal WC counterparts (Table 3).

**Table 2:** Rate comparison for all-cause hospitalization between BMI categories, by sex

	BMI category	*Crude rate values	Crude rate per 1000 person-year (95% CI)	Crude rate ratio (95% CI)	**Age adjusted rate ratio (95% CI)	†Multivariable adjusted rate ratio (95% CI)
**Men**	**Normal weight** **(n = 1389)**	1548/20,758	74.5 (68.3–80.7)	Reference	Reference	Reference
	**Overweight** **(n = 1737)**	2057/26,839	76.6 (71.5–81.7)	1.02 (0.96–1.09)	1.06 (0.95–1.16)	1.07 (0.97–1.18)
	**Obese** **(n = 601)**	803/9207	87.2 (78.1–96.3)	1.17 (1.07–1.27)	1.27 (1.11–1.42)	1.27 (1.12–1.43)
**Women**	**Normal** **(n = 999)**	886/15,889	55.7 (50.1–61.3)	Reference	Reference	Reference
	**Overweight** **(n = 1867)**	2192/29,562	74.1 (69.0-79.2)	1.33 (1.22–1.43)	1.19 (1.05–1.34)	1.17 (1.03–1.31)
	**Obese** **(n = 1609)**	2354/25,173	93.5 (87.0-99.9)	1.67 (1.55–1.81)	1.44 (1.27–1.61)	1.40 (1.23–1.56)

*Total number of hospitalization/number of person-years

** Rate ratio were estimated using negative binomial models adjusted for age

**†** Rate ratio were estimated using negative binomial models adjusted for age, education level, smoking status, marital status and physical activity level

**BMI**: body mass index; **CI**: confidence interval

**Normal weight**: BMI ≥ 18.5 to < 25.0 kg m^2^; **Overweight**: BMI ≥ 25.0 to < 30.0 kg m^2^; **Obese**: BMI ≥ 30.0 kg m^2^

**Table 3:** Rate comparison for all cause hospitalization between categories of WC, by sex

	WC	*Crude rate values	Crude rate per 1000 person-year (95% CI)	Crude rate ratio (95% CI)	**Age adjusted rate ratio (95% CI)	†Multivariable adjusted rate ratio (95% CI)
**Men**	**Normal WC** **(n = 2309)**	2472/35,573	69.4 (65.1–73.8)	Reference	Reference	Reference
	**High WC** **(n = 1418)**	1936/21,232	91.2 (84.8–97.4)	1.31 (1.23–1.39)	1.19 (1.08–1.29)	1.18 (1.08–1.29)
**Women**	**Normal WC** **(n = 2751)**	2709/44,303	61.1 (57.5–64.7)	Reference	Reference	Reference
	**High WC** **(n = 1724)**	2723/26,321	103.4 (96.7-110.1)	1.69 (1.60–1.78)	1.32 (1.20–1.44)	1.30 (1.18–1.41)

*Total number of hospitalization/number of person-years

** Rate ratio were estimated using negative binomial models adjusted for age

**†** Rate ratio were estimated using negative binomial models adjusted for age, education level, smoking status, marital status and physical activity level

**CI**: confidence interval; **WC**: waist circumference

**Normal WC**: WC < 95 cm; **High WC**: WC ≥ 95 cm

Table 4 shows the adjusted rates of all-cause hospitalizations by BMI and WC categories from NB regression models. Overall, the adjusted rates of all-cause hospitalizations were higher among obese men and women than their normal weight counterparts. Similarly, those with high WC had higher adjusted rates of all-cause hospitalizations than those with normal WC.

**Table 4:** Adjusted rates of all-cause hospitalization per 1000 person-years (95% CI) by baseline obesity status from negative binomial models: the TLGS study, 1999–2018

	BMI status	Men	Women
**Model 1**	Normal	64.9 (60.4–69.7)	57.9 (52.9–63.3)
	Overweight	68.8 (64.6–73.2)	69.4 (65.2–73.8)
	Obese	82.4 (74.5–91.2)	83.6 (78.5–89.1)
**Model 2**	Normal	63.6 (56.6–71.3)	62.0 (55.5–69.2)
	Overweight	68.1 (61.3–75.6)	72.8 (66.8–79.4)
	Obese	81.2 (71.5–92.1)	86.8 (80.0-94.3)
	**Waist circumference**		
**Model 1**	Normal WC	64.9 (61.3–68.6)	64.1 (60.8–67.5)
	High WC	77.2 (72.2–82.6)	84.9 (79.7–90.3)
**Model 2**	Normal WC	64.2 (57.9–71.2)	67.8 (62.4–73.5)
	High WC	76.2 (68.7–84.6)	87.8 (81.0-95.3)

Model 1 was adjusted for age

Model 2 was adjusted for age, marital status, educational level, smoking status and physical activity level

Adjusted rates were calculated using coefficients from negative binomial models for a person with the following characteristics: age = mean age, education = less than 6 years of schooling, smoking status = never smoker, marital status = married, PAL = low

**BMI**: body mass index; **PAL**: physical activity level; **WC**: waist circumference

**Normal weight**: BMI ≥ 18.5 to < 25.0 kg m^2^; **Overweight**: BMI ≥ 25.0 to < 30.0 kg m^2^; **Obese**: BMI ≥ 30.0 kg m^2^

**Normal WC**: WC < 95 cm; **High WC**: WC ≥ 95 cm

The pattern of higher adjusted rates of all-cause hospitalization in obese individuals and those with high WC, compared with their normal counterparts, remained present when we developed zero-inflated NB regression models (Supplementary Table 7).

**Fig. 1:**
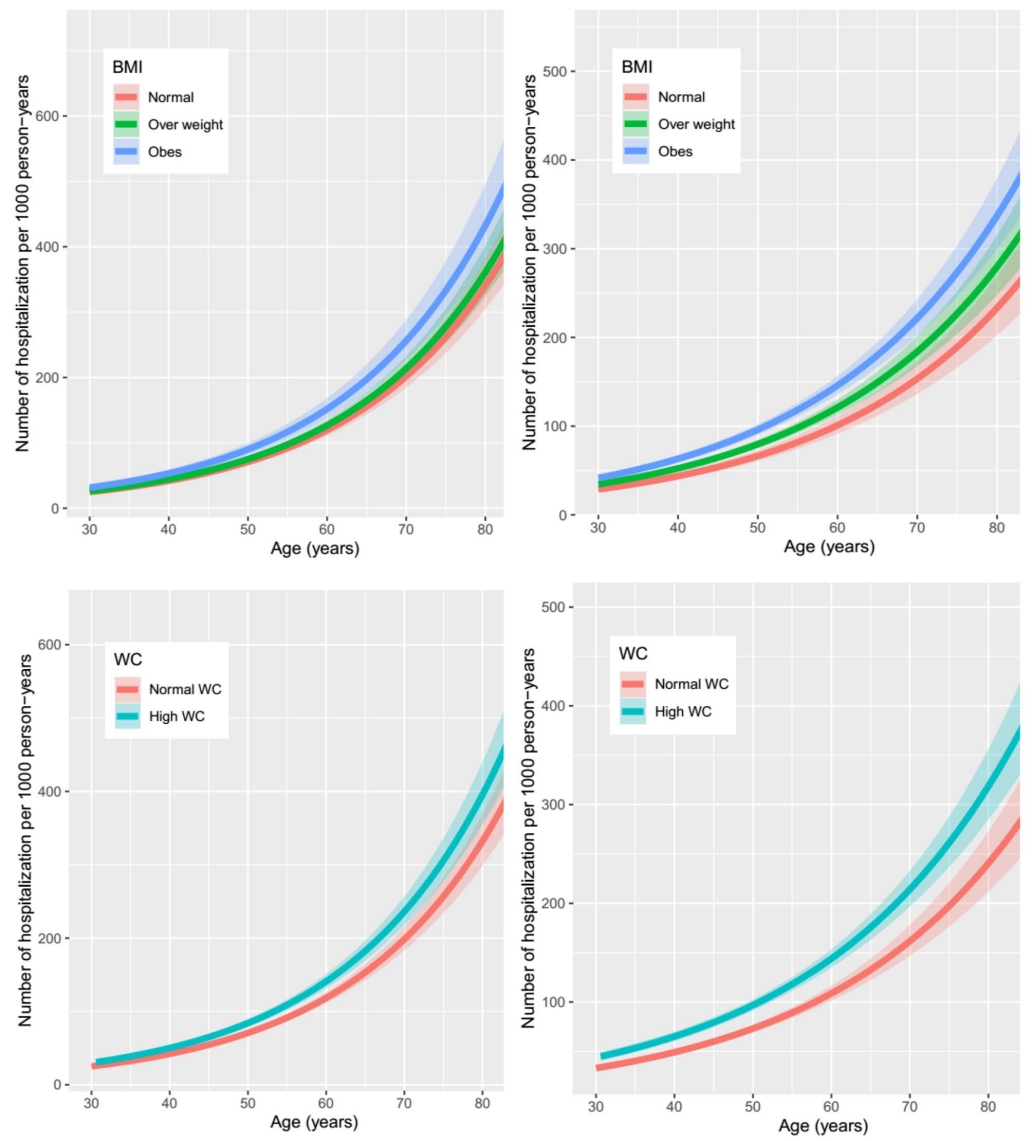
Adjusted rates of all-cause hospitalization per 1000 person-years by age and by baseline BMI and WC categories: the TLGS study, 1999-2018 **BMI**: body mass index; **WC**: waist circumference; **TLGS**: Tehran Lipid and Glucose Study

Figure 1 presents the adjusted rates of all-cause hospitalization by age and by baseline BMI and WC categories in men and women. The plots show that the effect of BMI and WC on the number of hospitalization is greater at older age. Adjusted rates among those who were older at baseline were higher than among those who were younger at baseline across BMI and WC categories.

## Discussion

In this cohort study of Iranian adults ≥ 30 years of age at baseline, we found that overweight in women and obesity in both sexes was associated with an increased rate of all-cause hospitalization over a median of 18 years of follow-up, even after adjustment for important confounders. We further recognized that central obesity was associated with higher rate of all-cause hospitalization in both sexes, independent of all confounders.

Our findings are largely consistent with indirect comparison studies, which have shown that obesity was associated with increased rates/risk of hospitalization, in either short or long-term [7, 12, 24–26]. One of these studies [12] examined the association between BMI status and number of all-cause and cause-specific hospitalizations among 15,355 adults from the Atherosclerosis Risk in Communities Study (ARIC). Other study [24] investigated the association between BMI and health services use and costs among US adults. Two other studies [25, 26] used data from a large-scale Australian cohort study to estimate the risk of hospital admission in relation to self-reported BMI. All the above mentioned studies found a positive association between obesity and hospitalization rate/risk.

In a study comparable to ours [13], but where participants were 65 to 100 years of age, overweight and obesity were associated with higher risk of hospitalizations in individuals aged 65 to 75 years; however, among people older than 75, overweight, and mild obesity were not associated with higher risk of hospitalizations. We did not perform analyses for different age groups; instead, we showed that the effect of high BMI and WC on the hospitalization rate was greater at older age.

None of the previous studies investigated the association between WC and hospitalization rate; however, we found a positive association between high WC and rate of hospitalization in both sexes.

In this study, we did not examine the effects of obesity indices on each primary causes of hospitalization, due to small number of cause specific hospitalization in our study sample.

However, we found that general adiposity in women and central adiposity in both sexes was associated with CHD-associated hospitalizations, with stronger impact among men than women. Consistent with this finding, Han et al. [12] found that overweight and obese adults had more hospitalizations for all of the CVD-related causes, osteoarthritis and back problems than normal weight adults. Other study [26] showed a significantly higher rate of hospitalization for diabetes, ischaemic heart disease, chest pain, diverticular disease, gallbladder disease, osteoarthritis, asthma, sleep apnoea and cellulitis in obese than normal weight adults.

Previous studies in our country have shown that the average BMI of Iranian adults has increased over the past two decades [27], and that about 60% of Iranian adults are overweight or obese [5]. On the other hand, in 2014, CHD contributed more than 16% of whole healthcare financial costs in Iran [28]. Moreover, healthcare costs has increased substantially in recent years [29], hence, it is expected that the economic cost of CHD to be much higher now, and will continue to rise for decades. Therefore, our findings suggest that overweight, not just obesity is also a substantial contributor to health service use and health care costs in the population, particularly among women, and the effective strategies for prevention and control of both general and central obesity is essential in order to improve population health and reduce healthcare costs.

This large prospective study provides the evidence to date for an association between obesity measures and the risk of all-cause hospitalization among population from MENA region. As epidemiologic studies have shown that BMI alone may not be a good measure of adiposity, we additionally used the WC as a simple measure of abdominal obesity in our analysis, hence, our results extend the literature by showing not only general obesity, but also central obesity is associated with a higher risk of hospitalization with a significant excess rates later in life. It is also a strength that height and weight were objectively measured rather than from self-report. Some previous studies have examined the association between self-reported body weight and hospital admission [25, 26, 30]. It is well established that self-reports tend to bias reports of weight and height [25, 31]. Several previous studies have estimated the effect of BMI on the first hospital admission [25, 26, 32]. In this study, the effect of obesity on the total number of hospitalizations was examined; thus, we were able to quantify the cumulative burden of increased hospitalization associated with obesity indices.

There may be several limitations to our study. First, we did not have data about insurance status, a factor that strongly affect the hospitalization rate among participants [33]. However, a study in Iran showed that health insurance coverage was extended to all rural residents during 2004–2013 [34].

Second, we did not stratify analysis for each specific cause of hospitalization, due to the small number of cases for several causes. However, the high crude rate of hospitalization due to CHD, cancer, and DM complications among obese and those with high WC is concerning and suggests that increased efforts are needed to reduce the burden of preventable hospitalization among persons with general and central obesity. Third, our study sample was selected from the metropolitan of Tehran which may limit the generalizability of the findings to the country’s rural areas. Finally, we did not account for the change in risk factors during follow-up period.

## Conclusion

This study showed considerable excess hospitalization rate associated with general and central obesity in both sexes. Our findings suggest that among the study population, the impact of successful weight management on the hospitalization is likely to be larger in women, and also in older than younger individuals. Our findings have implications for obese and overweight individuals as well as for policy-makers which aims to improve population health and to manage direct medical cost and resource use burden imposed on the health care system in our country.

## Electronic supplementary material

Below is the link to the electronic supplementary material.

Supplementary Material 1

## Data Availability

The datasets analysed during the current study are available from the corresponding author on reasonable request.

## List of Abbreviations

BMI: Body mass index
WC: Waist circumference
IRRs: Incidence rate ratios
GBD: Global Burden of Disease
MENA: Middle East and North Africa
TLGS: Tehran lipid and glucose study
NCD: Non-communicable diseases
PAL: Physical activity level
MET: Metabolic equivalent task minutes
CVD: Cardiovascular disease
ICD-10: International Classification of Diseases, 10th revision
CHD: Coronary heart disease
DM: Diabetes mellitus
ANOVA: Analysis of variance
NB: Negative binomial
IQR: Interquartile range
