# Supplementary material for: The impact of general and central obesity for all-cause hospitalization among Iranian adults: a 20 year follow-up-results from the TLGS cohort
Source: BMC Public Health. 2023 May 18;23:903. doi: 10.1186/s12889-023-15851-0 (PMC10193790; doi:10.1186/s12889-023-15851-0)
Supplement: Supplementary file 1 — Supplementary Material 1 [file 12889_2023_15851_MOESM1_ESM.docx]

**SUPPLEMENTARY MATERIALS**

9558 participants aged ≥ 30 years,

7927 recruited from Phase 1 (1999-2002)

, and 1631 recruited from Phase 2 (2002-2005)

**Exclusion:**

People with no follow-up data, n=879

People with missing data on BMI and WC at baseline, n=276

Those with BMI <18.5 kg/m^2^, n=109

Those with missing data on study covariates at baseline, n=92

Final study sample,

n= 8202 (3727 men)

**Supplementary Figure 1:** Flowchart of sample selection for the study, the TLGS study, 1999-2018

**BMI**: body mass index; **TLGS**: Tehran Lipid and Glucose Study

| **Supplementary Table 1:** Categories of hospitalization causes by ICD-10 codes | | | | | | | | |
| --- | --- | --- | --- | --- | --- | --- | --- | --- |
| **CHD** | **Stroke** | **Infectious disease** | **Respiratory disease** | **DM complication** | **HTN complication** | **Neoplasm** | **Trauma** | **Others** |
| Fatal MI  I22 | Fatal stroke  I64 | Sepsis  A40-41 | Asthma  J45-46 | Diabetic nephropathy  E11.2 | Malignant HTN  I10.10 | Breast cancer  C50 | Accident  V01-99 | Benign tumors  D10-36 |
| Non-fatal MI  I21 | Non-fatal stroke  I63 | Pneumonia  J12-18 | Chronic bronchitis  J40-42 | Diabetic foot ulcer  E11.5 | HTN-related cardiac disease  I11 | Lung cancer  C34 | Fractures  T12-14.2 | Un-classified non-coronary disease  I30-52 |
| Definite CHD  I25 |  |  | Emphysema  J43 | Hypoglycemia  E16.0,1,2 | HTN crisis  I16.0,1,9 | Gastrointestinal cancer  C15-21,23-24,26 |  | Poisoning  T51-65 |
| Sudden cardiac death  I46 |  |  | Bronchiectasis  J47 | Diabetic ketoacidosis  E11.1 | HTN complication  I12-13 | Prostate cancer  C61 |  | Drug overdoses  T36-50 |
| Heart failure  I50 |  |  | COPD  J44 | Hyperosmolar state  E11.00 | HTN in pregnancy  O.13 | Bladder cancer  C67 |  | Unclassified disease |
| Unstable angina  I20 |  |  |  | Other DM complication  E11.3,4,6,7,8 |  | Uterus cancer  C54-55 |  |  |
| Peripheral artery disease  I73 |  |  |  | DM metabolic control  E70-90 |  | Liver cancer  C22 |  |  |
|  |  |  |  | GDM  O.24 |  | Pancreas cancer  C25 |  |  |
|  |  |  |  | Metabolic surgery for patients with diabetes  Z.98 |  | Other cancers  C00-97(excluding above) |  |  |
| **ICD-10;** International Classification of Diseases 10th Revision; **CHD**: coronary heart disease; **MI**: myocardial infarction; **COPD**: Chronic obstructive pulmonary disease; **DM**: diabetes mellitus; **GDM**; gestational diabetes mellitus; **HTN**: hypertension | | | | | | | | |

| **Supplementary Table 2:** Baseline characteristics of participants and non-participants: the TLGS study, 1999-2018 | | | |
| --- | --- | --- | --- |
|  | **Participants**  **(n=8202)** | **Non-participants (n=1356)** | **P value** |
| **Continuous variables** |  |  |  |
| Age (years) | 47.4 (12.3) | 48.1 (13.8) | 0.065 |
| BMI (kg/m^2^) | 27.6 (4.4) | 26.5 (5.3) | <0.001 |
| Waist circumference (cm) | 91.1 (11.2) | 88.3 (12.9) | <0.001 |
| **Categorical variables** |  |  |  |
| **Smoking (%)** |  |  |  |
| Never | 6099 (74.4) | 803 (69.5) | <0.001 |
| Past | 731 (8.9) | 104 (9.0) |  |
| Current | 1372 (16.7) | 249 (21.5) |  |
| **Marital status (%)** |  |  |  |
| Single | 318 (3.9) | 54 (4.0) | 0.478 |
| Married | 7267 (88.6) | 1182 (87.6) |  |
| Widowed/divorced | 617 (7.5) | 114 (8.4) |  |
| **Educational level (%)** |  |  |  |
| <6 years | 3300 (40.2) | 571 (42.8) | 0.214 |
| 6-12 | 3925 (47.9) | 610 (45.7) |  |
| ≥12 | 977 (11.9) | 154 (11.5) |  |
| **Physical activity level** |  |  |  |
| High | 2499 (30.5) | 291 (25.5) | 0.001 |
| Low | 5703 (69.5) | 849 (74.5) |  |
| **BMI**: body mass index; **TLGS**: Tehran Lipid and Glucose Study | | | |

| **Supplementary Table 3:** Crude rates of all-cause and cause-specific hospitalization per 1000 person-years in total participants by BMI categories: the TLGS study, 1999-2018 | | | | | | |
| --- | --- | --- | --- | --- | --- | --- |
|  | **Normal**  **(n=2388)** | | **Overweight**  **(n=3604)** | | **Obese**  **(n=2210)** | |
|  | **n** | **Crude rate per 1000 person-year (95% CI)** | **n** | **Crude rate per 1000 person-year (95% CI)** | **n** | **Crude rate per 1000 person-year (95% CI)** |
| All-cause hospitalizations | 2434 | 73.4 (68.5-78.3) | 4249 | 79.7 (75.8-83.7) | 3157 | 95.1 (89.5-100.6) |
| CHD | 650 | 20.2 (17.8-22.6) | 1242 | 23.5 (21.5-25.5) | 815 | 25.1 (22.2-27.8) |
| Stroke | 80 | 2.4 (1.8-3.1) | 177 | 3.5 (2.9-4.2) | 122 | 3.7 (2.9-4.5) |
| Cancer | 222 | 7.8 (6.1-9.5) | 300 | 6.4 (5.2-7.6) | 195 | 6.6 (5.1-8.2) |
| HTN complication | 37 | 1.1 (0.6-1.5) | 81 | 1.4 (1.1-1.8) | 81 | 2.3 (1.7-2.9) |
| DM complication | 74 | 2.5 (1.6-3.4) | 139 | 2.7 (2.1-3.4) | 165 | 4.8 (3.8-5.8) |
| Infection | 104 | 3.1 (2.3-3.7) | 120 | 2.2 (1.7-2.7) | 107 | 3.2 (2.5-3.9) |
| Respiratory | 63 | 2.2 (0.5-3.9) | 22 | 0.4 (0.1-0.6) | 30 | 0.9 (0.5-1.3) |
| Traumatic | 108 | 2.9 (2.3-3.5) | 197 | 3.5 (2.9-4.1) | 107 | 3.1 (2.4-3.7) |
| Others | 1096 | 30.3 (28.1-32.5) | 1971 | 35.3 (33.3-37.3) | 1535 | 44.8 (41.8-47.8) |
| **BMI:** body mass index; **CHD**: coronary heart disease; **HTN**: hypertension; **DM**: diabetes mellitus; **TLGS**: Tehran Lipid and Glucose Study | | | | | | |

| **Supplementary Table 4:** Crude rates of all-cause and cause specific hospitalization per 1000 person-years in total participants by WC categories: the TLGS study, 1999-2018 | | | | |
| --- | --- | --- | --- | --- |
|  | **Normal WC**  **(n=5060)** | | **High WC**  **(n=3142)** | |
|  | **n** | **Crude rate per 1000 person-year (95% CI)** | **n** | **Crude rate per 1000 person-year (95% CI)** |
| All cause hospitalizations | 5184 | 64.8 (62.1-67.6) | 4659 | 97.9 (93.2-102.6) |
| CHD | 1335 | 16.7 (15.4-18.0) | 1372 | 28.8 (26.3-31.3) |
| Stroke | 169 | 2.1 (1.7-2.4) | 210 | 4.4 (3.6-5.1) |
| Cancer | 429 | 5.3 (4.5-6.2) | 288 | 6.0 (4.9-7.1) |
| HTN complication | 93 | 1.2 (0.8-1.5) | 106 | 2.2 (1.7-2.7) |
| DM complication | 148 | 1.8 (1.4-2.2) | 230 | 4.8 (3.9-5.7) |
| Infection | 165 | 2.1 (1.7-2.4) | 166 | 3.5 (2.9-4.1) |
| Respiratory | 70 | 0.9 (0.3-1.5) | 45 | 0.9 (0.6-1.2) |
| Traumatic | 216 | 2.7 (2.3-3.1) | 196 | 4.1 (3.4-4.7) |
| Others | 2556 | 31.9 (30.4-33.5) | 2046 | 43.0 (40.5-45.4) |
| **WC:** waist circumference**; CHD**: coronary heart disease; **HTN**: hypertension; **DM**: diabetes mellitus; **TLGS**: Tehran Lipid and Glucose Study | | | | |

| **Supplementary Table 5:** Crude rates of all-cause and cause specific hospitalization per 1000 person-years in men and women by baseline BMI: the TLGS study, 1999-2018 | | | | | | | | | | | | | |
| --- | --- | --- | --- | --- | --- | --- | --- | --- | --- | --- | --- | --- | --- |
|  | **Men**  **n=3727** | | | | | |  | **Women**  **n=4475** | | | | | |
|  | **Normal**  **(n=1389)** | | **Overweight**  **(n=1737)** | | **Obese**  **(n=601)** | |  | **Normal**  **(n=999)** | | **Overweight**  **(n=1867)** | | **Obese**  **(n=1609)** | |
|  | ***n** | ****Crude rate**  **(95% CI)** | **n** | **Crude rate**  **(95% CI)** | **n** | **Crude rate**  **(95% CI)** |  | **n** | **Crude rate**  **(95% CI)** | **n** | **Crude rate**  **(95% CI)** | **n** | **Crude rate**  **(95% CI)** |
| All-cause | 1548 | 74.5  (68.3-80.7) | 2057 | 76.6  (71.5-81.7) | 803 | 87.2  (78.1-96.3) |  | 886 | 55.7  (50.1-61.3) | 2192 | 74.1  (69.0-79.2) | 2354 | 93.5  (87.0-99.9) |
| CHD | 480 | 23.1  (20.1-26.1) | 749 | 27.9  (25.1-30.7) | 286 | 31.1  (25.3-36.7) |  | 170 | 10.6  (8.1-13.2) | 493 | 16.6  (14.3-18.9) | 529 | 21.0  (18.0-24.0) |
| Stroke | 61 | 2.9  (2.1-3.7) | 109 | 4.1  (3.1-5.0) | 51 | 5.5  (3.6-7.4) |  | 19 | 1.1  (0.5-1.8) | 68 | 2.3  (1.6-2.9) | 71 | 2.8  (2.0-3.5) |
| Cancer | 139 | 6.7  (4.9-8.4) | 152 | 5.6  (4.3-6.9) | 40 | 4.3  (2.4-6.2) |  | 83 | 5.2  (3.4-7.0) | 148 | 5.0  (3.5-6.4) | 155 | 6.1  (4.5-7.8) |
| HTN | 15 | 0.7  (0.2-1.1) | 22 | 0.8  (0.4-1.1) | 12 | 1.3  (0.4-2.1) |  | 22 | 1.3  (0.6-2.0) | 59 | 1.9  (1.3-2.6) | 69 | 2.7  (1.9-3.5) |
| DM | 45 | 2.1  (1.1-3.1) | 55 | 2.0  (1.1-2.8) | 33 | 3.5  (2.1-4.9) |  | 29 | 1.8  (0.7-2.9) | 84 | 2.8  (1.9-3.7) | 132 | 5.2  (3.9-6.4) |
| Infection | 74 | 3.5  (2.5-4.5) | 57 | 2.1  (1.4-2.7) | 30 | 3.2  (1.9-4.5) |  | 30 | 1.8  (1.1-2.5) | 63 | 2.1  (1.4-2.8) | 77 | 3.1  (2.2-3.8) |
| Respiratory | 54 | 2.6  (0.4-4.7) | 12 | 0.4  (0.1-0.7) | 8 | 0.8  (0.1-1.5) |  | 9 | 0.5  (0.01-1.1) | 10 | 0.3  (0.07-0.6) | 22 | 0.8  (0.4-1.3) |
| Traumatic | 79 | 3.8  (2.9-4.6) | 86 | 3.2  (2.4-3.9) | 38 | 4.1  (2.6-5.5) |  | 29 | 1.8  (1.1-2.5) | 111 | 3.7  (2.9-4.5) | 69 | 2.7  (1.9-3.4) |
| Others | 601 | 28.9  (25.9-31.9) | 815 | 30.3  (27.7-32.9) | 305 | 33.1  (28.3-37.9) |  | 495 | 31.1  (27.8-34.4) | 1156 | 39.1  (36.2-41.9) | 1230 | 48.8  (45.2-52.4) |
| ***** Total number of hospitalization  ****** Crude rate per 1000 person-year (95% CI)  **BMI:** body mass index; **CI:** confidence interval; **CHD**: coronary heart disease; **HTN**: hypertension; **DM**: diabetes mellitus; **TLGS**: Tehran Lipid and Glucose Study | | | | | | | | | | | | | |

| **Supplementary Table 6:** Crude rates of all-cause and cause specific hospitalization per 1000 person-years in men and women by baseline WC categories: the TLGS study, 1999-2018 | | | | | | | | | |
| --- | --- | --- | --- | --- | --- | --- | --- | --- | --- |
|  | **Men**  **n=3727** | | | |  | **Women**  **n=4475** | | | |
|  | **Normal WC**  **(n=2309)** | | **High WC**  **(n=1418)** | |  | **Normal WC**  **(n=2751)** | | **High WC**  **(n=1724)** | |
|  | ***n** | ****Crude rate**  **(95% CI)** | ***n** | ****Crude rate**  **(95% CI)** |  | ***n** | ****Crude rate**  **(95% CI)** | ***n** | ****Crude rate**  **(95% CI)** |
| All-cause | 2472 | 69.4 (65.1-73.8) | 1936 | 91.2 (84.8-97.4) |  | 2709 | 61.1 (57.5-64.7) | 2723 | 103.4 (96.7-110.1) |
| CHD | 804 | 22.6 (20.3-24.8) | 711 | 33.4 (29.7-37.1) |  | 531 | 11.9 (10.4-13.5) | 661 | 25.1 (21.8-28.3) |
| Stroke | 105 | 2.9 (2.3-3.5) | 116 | 5.4 (4.1-6.7) |  | 64 | 1.4 (1.0-1.8) | 94 | 3.5 (2.7-4.4) |
| Cancer | 204 | 5.7 (4.5-6.9) | 127 | 5.9 (4.4-7.5) |  | 225 | 5.1 (3.8-6.2) | 161 | 6.1 (4.5-7.6) |
| HTN | 25 | 0.7 (0.3-1.03) | 24 | 1.1 (0.6-1.6) |  | 68 | 1.5 (1.1-1.9) | 82 | 3.1 (2.2-3.9) |
| DM | 69 | 1.9 (1.1-2.6) | 64 | 3.0 (2.0-3.9) |  | 79 | 1.7 (1.2-2.3) | 166 | 6.3 (4.8-7.7) |
| Infection | 93 | 2.6 (1.9-3.2) | 68 | 3.2 (2.2-4.1) |  | 72 | 1.6 (1.1-2.1) | 98 | 3.7 (2.8-4.5) |
| Respiratory | 56 | 1.6 (0.3-2.9) | 18 | 0.8 (0.3-1.3) |  | 14 | 0.3 (0.1-0.5) | 27 | 1.0 (0.5-1.4) |
| Traumatic | 124 | 3.5 (2.8-4.1) | 79 | 3.7 (2.8-4.5) |  | 92 | 2.1 (1.6-2.5) | 117 | 4.4 (3.4-5.4) |
| Others | 992 | 27.8 (25.6-30.1) | 729 | 34.3 (31.1-37.5) |  | 1564 | 35.3 (33.0-37.5) | 1317 | 50.0 (46.5-53.5) |
| ***** Total number of hospitalization  ****** Crude rate per 1000 person-year (95% CI)  **WC:** waist circumference; **CI:** confidence interval; **CHD**: coronary heart disease; **HTN**: hypertension; **DM**: diabetes mellitus; **TLGS**: Tehran Lipid and Glucose Study | | | | | | | | | |

| **Supplementary Table 7:** Adjusted rates of all-cause hospitalization per 1000 person-years (95% CI) by baseline obesity status from zero-inflated negative binomial models: the TLGS study, 1999-2018 | | | |
| --- | --- | --- | --- |
|  | **Men** | **Women** |  |
|  |  |  |  |
| **BMI status** |  |  |  |
| Normal | 75.6 (66.2-86.3) | 62.4 (55.1-70.7) |  |
| Overweight | 79.1 (70.2-89.1) | 73.2 (65.9-81.3) |  |
| Obese | 93.3 (81.5-107.0) | 87.2 (78.9-96.5) |  |
|  |  |  |  |
| **Waist circumference** |  |  |  |
| Normal WC | 75.8 (67.2-85.4) | 68.3 (62.9-74.1) |  |
| High WC | 88.3 (78.5-99.3) | 88.4 (81.4-96.3) |  |
| Zero-inflated negative binomial model was adjusted for age, marital status, educational level, smoking status and physical activity level  Adjusted rates were calculated using coefficients from zero-inflated negative binomial model for a person with the following characteristics: age=mean age, education= less than 6 years of schooling, smoking status= never smoker, marital status= married, PAL= low  **BMI:** body mass index; **PAL**: physical activity level; **WC**: waist circumference  **Normal weight:** BMI ≥18.5 to <25.0 kg m^2^; **Overweight**: BMI ≥25.0 to <30.0 kg m^2^; **Obese**: BMI≥30.0 kg m^2^  **Normal WC**: WC <95 cm; **High WC**: WC ≥ 95 cm | | | |
